# Identify Melatonin as a Novel Therapeutic Reagent in the Treatment of 1-Bromopropane(1-BP) Intoxication

**DOI:** 10.1097/MD.0000000000002203

**Published:** 2016-01-22

**Authors:** Yongpeng Xu, Shuo Wang, Lulu Jiang, Hui Wang, Yilin Yang, Ming Li, Xujing Wang, Xiulan Zhao, Keqin Xie

**Affiliations:** From the Institute of Toxicology, School of Public Health, Shandong University, Jinan, Shandong, PR China.

## Abstract

1-Bromopropane (1-BP) has been used as an alternative for fluoride compounds and 1-BP intoxication may involve lung, liver, and central neural system (CNS). Our previous studies showed that 1-BP impaired memory ability by compromising antioxidant cellular defenses. Melatonin is a powerful endogenous

antioxidant, and the objective of this study was to explore the therapeutic role of melatonin in the treatment of 1-BP intoxication. Rats were intragastrically treated with 1-BP with or without melatonin, and then sacrificed on 27th day after 1-BP administration. The Morris water maze (MWM) test was used to evaluate the spatial learning and memory ability of the experimental animals, and NeuN staining was performed to assess neuron loss in hippocampus. We found that rats treated with 1-BP spent more time and swam longer distance before landing on the hidden platform with a comparable swimming speed, which was markedly mitigated by the pretreatment with melatonin in a concentration-dependent manner. In addition, 1-BP-induced notable decrease in neuron population in hippocampus by promoting apoptosis, and melatonin pretreatment attenuated those changes in brain. The GSH/GSSG ratio was proportionately decreased and heme oxygenase 1 was increased in the rats exposed to 1-BP (Figure 6), and administration of melatonin restored them. Meanwhile, MDA, the level of lipid peroxidation product, was significantly increased upon exposed to 1-BP, which was significantly attenuated by melatonin pretreatment, indicating that administration of 1-BP could interfere with redox homeostasis of brain in rat, and such 1-BP-induced biomedical changes were reversed by treatment with melatonin.

We conclude that treatment with melatonin attenuates 1-BP-induced CNS toxicity through its ROS scavenging effect.

## INTRODUCTION

As an alternative for fluoride compounds, 1-bromopropane (1-BP) has been widely used as composite ingredients for a variety of raw materials for agrochemical, medicines, organic compounds, detergents, and solvents for its characteristics such as low inflammability, volatile property, and particularly low possibility to injure the ozone layer. However, accumulative evidence from animal experiments as well as epidemiologic studies showed 1-BP might be toxic to human body.^1^ The toxicity of 1-BP on injuries to the lungs and liver ^2^ and inhibition of the central nervous system^3^ have been studied. Recently, Yu et al^4^ conducted an animal experiment and found that exposure to 1000 ppm of 1-BP 8 h per day for consecutive 7 weeks might cause lower nerve conduction velocity, disorder of gait, and loss of weight, whereas Kim et al^5^ found that repeated inhalation of 1800 ppm of 1-BP for 8 weeks resulted in weight reduction, but no obvious findings were detected in histological examination or in a variety laboratory tests of urine and blood.

It has been shown that exposure to 1-BP, particularly at high concentrations (1000 ppm), may cause morphological alternation in microglia in rat brains.^6^ Moreover, the exposure to 1-BP triggers a range of biochemical events that lead to oxidative stress (formation of ROS), which might be the underlying mechanism of neurotoxicity of 1-BP.^6^ Normally, productions of ROS and reactive nitrogen species (RNS) are initiated upon cellular stress, whereas antioxidants remove them rapidly to prevent cell death or dysfunction. If such balance between oxidants and antioxidants gets compromised, the cellular homeostasis is disturbed, which might be responsible for the development of a wide range of human neurodegenerative diseases such as Parkinson's disease.^7–11^

Originally regarded as a secretory product by the pineal gland, melatonin (*N*-acetyl-5-methoxytryptamine, melatonin) has a fundamental function in the neuro-immuno-endocrine system.^12^ Benign effective antioxidant, melatonin feeds off directly peroxynitrite anion (ONOO–) and _OH.^13^ In addition, melatonin decreases free radicals by activating the functions of enzymes associated with antioxidative defence.^14–16^ Moreover, attention has been paid to the role of melatonin to regulate apoptosis; it restrains apoptosis and necrosis of cells in the kidney,^17^ myocardium,^18^ brain,^19^ liver, ^20^ and pancreas.^21^ Earlier evidence demonstrated that administration of melatonin decreased the proportion of injured spermatozoa caused by exposure to 1-BP in man.^22^ Hence, whether melatonin exhibits its effects to protect reproductive toxicity induced by 1-BP is of great interest. Based on the above-mentioned evidence, we hypothesize that melatonin treatment might function as an antidote for 1-BP intoxication by fighting against oxidants. To test it, we established animal model of 1-BP intoxication, which were subsequently treated with melatonin. Memory ability and spatial learning were evaluated, and biomedical lab tests and oxidant status were determined in those animals in comparison with the controls.

## MATERIALS AND METHODS

Establishment of animal model of 1-BP intoxication and administration of melatonin 90 adult male Sprague-Dawley rats (SD rats) were obtained from Beijing Vital River Laboratory Animal Technology Co., Ltd, and maintained in plastic cages. All SD rats were male with weight between 270 g and 280 g. The conditions were controlled at room temperature (22 ± 2°C) and relative humidity of 50% to 60% under 12 h light and 12 h dark. The rats were fed with tap water and standard rat diet. The rats were maintained for 5 days and then randomly assigned to 6 groups, including control group, 1-BP 600 mg/kg group bw, 1-BP 600 mg/kg bw + melatonin 2.5 mg/kg bw, 1-BP 600 mg/kg bw + melatonin 5 mg/kg bw, 1-BP 600 mg/kg bw + melatonin 10 mg/kg bw, and melatonin 10 mg/kg bw. No difference was noted among each group regarding the weight of the SD rats (data not shown). The exposure concentrations were determined according to the previous work done in our lab, in which toxicity on the central and peripheral nervous systems was observed in rats after treatment with 1-BP.^23^ Rats in treatment groups were intragastrically administered with 1-BP which was dissolved in corn oil each day for 27 consecutive days, and 1 h after administration of 1-BP, melatonin was given intraperitoneally. The rats in the control group were administered with the same volume of corn oil. The cognitive function of rats was assessed between D23 and D27 of the experiment using the MWM test. Subsequently, rats were subject to anesthesia with over-dose pentobarbital sodium and sacrificed 24 h following completion of the MWM test. The hippocampus were rapidly removed, frozen in liquid nitrogen, and maintained at −80°C until use.

### Ethics Statement

The approval of study design and procedures was obtained from the Animal Experiments Ethics Committee in Shandong University (Approval Number: 20140807) in concordance with the Guideline for Care and Use of Laboratory Animals by NIH.

### Morris Water Maze (MWM) Test

The spatial learning and memory ability was evaluated using the MWM test.^24^ The maze included a circular tank (1.80 m diameter, 0.6 m depth) with black interior containing opaque water kept at a temperature of 25 ± 1°C. The animal movement and swimming type in the maze was monitored and recorded for using a computerized monitoring system (Huaibei ZhengHua Biological Instrument Equipment Co., Ltd, Suixi, China) equipped with video monitoring software a video camera directly attached above the maze manufactured by Huaibei ZhengHua Biological Instrument Equipment Co., Ltd (Suixi, China).

### Spatial Navigation Test

Their distance traveled (length of the swimming path traveled to find the platform) and escape latency (time to position of the hidden platform) were tacked to determine the spatial learning of rats using the Spatial navigation test. During the test, animals were slowly placed to the water and their heads faced against the pool wall starting from aperipheral pseudorandom positions (W, E, N, or S) to position the platform hidden at 1 to 2 cm lower than the water surface for 120 s. Each rat was subject to 4 s swimming tests beginning randomly from 4 positions each day for consecutive 4 days. The distance traveled and escape latency were recorded for each test. After each test, rats were placed in a cage for the intertrial interval of a 30 s. If the rat could not locate the platform within 120 s, it was slowly instructed to the platform and permitted to rest for 15 s, and then the record of escape latency time of 120 s was restarted. Data from the 4 starting points are pooled together for statistical data of each test session. The ability of spatial learning was determined using the search method designed by Graziano et al^25^ during assessment of the individual swimming path. Data were indicated as the percentage of various search type characterized by the rats in a session of day 5. Data processing: live images of the swimming animals were collected via a digital video camera, shooting toward the pool, connected to a computer-controlled tracking system and sampled in time series at a rate of 7 frames/s. Each pair of coordinates represented the position of the center of gravity (object) of the rat's image. The sets of data acquired for each trial were stored on disk as single tracks’ files. Data were expressed as the portion of different searching pattern manifested by the rats in all 4 days. Four representative learning patterns were qualitatively analyzed in the present study. Thigmotaxis (rats swim almost exclusively in the periphery) and random searching (the moving trajectories are no longer circular but jagged with sudden changes in direction and velocity) were considered as the ineffective ways, and approaching target (rats adjust its swimming trajectory while approaching the platform) and direct finding (rats swim fast and straight from the starting point to the platform) as effective ways. Determination of the ratio of reduced glutathione (GSH) to oxidized glutathione (GSSG).

A GSH contest determination kit was obtained from Beyotime (Nantong, China), and the ratio of GSH to GSSG was measured following the instruction provided by the manufacturer.

### Determination of Malondialdehyde (MDA) Content

A total of 2 mL of 10 mM phosphate buffer (pH 7.4) was used to homogenize the left cerebral cortex tissue. The tissue was centrifuged at 12,000 g for 20 min. Following centrifugation, the MDA contents in the supernatant were determined using the spectrophotometrical method with the kits purchased from Beyotime (Nantong, China). The Bradford method was used to determine the protein levels. The level of MDA was indicated as nmol/g protein.

### Western Blot Analysis

The tissue samples were washed twice with phosphate buffered saline (PBS), homogenized, and lysed in the RIPA buffer (Upstate, Billerica, MA) for 20 min at 4 °C. Lysates were centrifuged at 12,000 g at 4°C for 12 min and we separated supernatants for western analysis. SDS-PAGE (10% gel) was used to separated equal amount (40 mg) of cell lysates, followed by transferring them to polyvinylidene membranes (Millipore, Billerica, MA), and then probed them with antibodies against proteins of interest, including anti-Heme oxygenase1 antibody (1:1000, room temperature for 2 h, Santa Cruz Biotechnology, Santa Cruz, CA) and β-actin antibody (1:10000, room temperature for 1 h, Santa Cruz Biotechnology, Santa Cruz, CA). Appropriate horse radish peroxidase-conjugated secondary antibodies (Santa Cruz Biotechnology, Santa Cruz, CA) were adopted to incubate blots. Enhanced chemiluminescence (ECK) kit (Amersham ECL detection system, GE Healthcare) was used to visualize bound antibodies, and the relative density of the target bands was determined by using densitometry analysis.

### Neuron Loss Estimation

Rat brains stored in 30% sucrose for 3 days were sliced into 40-μm thick coronal sections on a freezing microtome. Four sections per rat were selected and stored in 0.1 M PBS for the evaluation of neuron loss by staining with NeuN, a neuronal marker. Washed with PBS, the sections were incubated with 1% H_2_O_2_ for 15 min, and blocked with 1% bovine serum albumin (BSA), 4% normal goat serum, and 0.4% TritonX-100 in PBS for 30 min at room temperature. Next, the sections were incubated with primary antibody (anti-NeuN antibody, 1:500 dilution; ab177487; Abcam, Cambridge, UK) at 4°C overnight. Then the sections were rinsed with PBS twice for 15 min and incubated in avidin/biotinylated peroxidase complex (Vector Laboratories, Burlingame, CA) for 1 h at room temperature. Sections were then rinsed with PBS thrice for 10 min and stained with a solution of 0.05% diaminobenzidine (DAB) and 0.03% H_2_O_2_. Subsequently, the sections were mounted on slides, air-dried, dehydrated in graded alcohol, cleared in xylene, and coverslipped. The neuron loss was microscopically evaluated and ImageJ software (National Institutes of Health) was used to analyze the images.

### TUNEL Procedure

Apoptosis was assessed by detecting DNA fragmentation through TUNEL staining. TUNEL staining for apoptotic neurons in the hippocampus were performed in accordance with instructions of the manufacturer (Roche Corporation, Germany). The specimens in each group were observed using a microscope and in at least 6 random fields of high-power field (×100).

### Statistical Analysis

Data were indicated as mean ± SE. One-way ANOVA followed by Dunnett's test was used to compare differences between the treatment and control groups. Pearson's analysis was used to evaluate the correlation. Each exposure level and parameter in a model were designated as independent value and dependent value, respectively. Statistical significance was indicated by *P* < 0.05.

## RESULTS

1. Establishment of the animal model for 1-BP intoxication

To establish the animal model for 1-BP intoxication, Sprague-Dawley rats were treated with 1-BP (600 mg/kg) as described in the methods section. Upon initiation of the administration of 1-BP, irritability was observed in the majority of the animals. One week after the initiation of the treatment, more symptoms including slow response, drowsiness, and sluggishness were recorded without any unexpected death.

2. Treatment with melatonin mitigated 1-BP caused impairment of memory ability and spatial learning.

**FIGURE 1:**
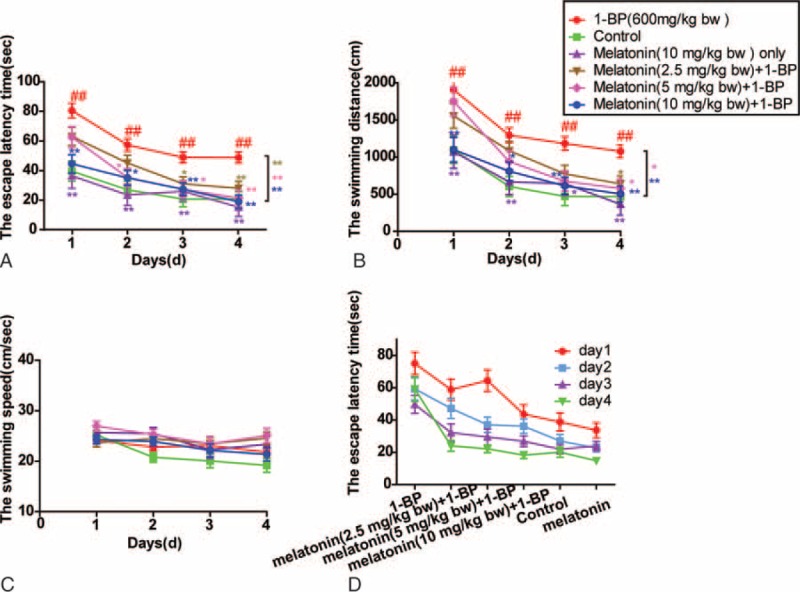
Effects of melatonin on 1-bromopropane (1-BP)-treated SD rats of spatial learning ability in Morris Water Maze (MWM). Data were displayed as mean ± SEM (n=12). (A) The escape latency time in SD rats. (B) The swimming distance in SD rats. (C) The swimming speed of SD rats. (D) The changes of the escape latency time in the groups on Day 1–4. ∗*P* < 0.05, ∗∗*P* < 0.01 versus 1-BP group; ^##^*P* < 0.01 versus control group. 1-BP = 1-bromopropane, MWM = Morris Water Maze, SD rats = Sprague-Dawley rats, SEM = Standard Error of Mean.

**FIGURE 2:**
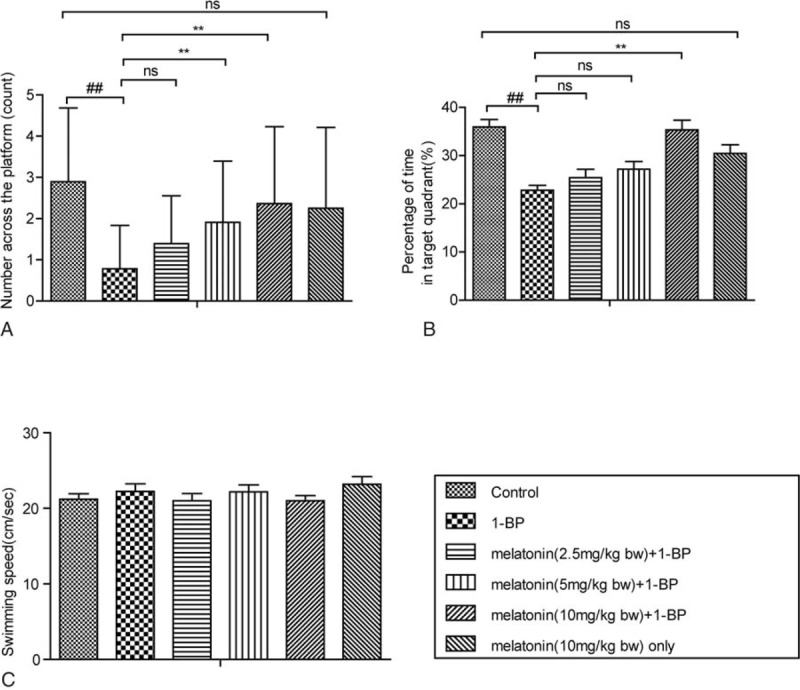
Effects of melatonin on 1-bromopropane (1-BP) influenced SD rats of spatial memory ability in Morris Water Maze (MWM). Data were shown as mean ± SEM (n = 12). (A) The number across the platform in SD rats. (B) The percentage of time in target quadrant in SD rats. (C) The swimming speed of SD rats. ∗*P* < 0.05, ∗∗*P* < 0.01, ^ns^*P* > 0.05 versus 1-BP group; ^##^*P* < 0.01 versus control group. 1-BP = 1-bromopropane, MWM = Morris Water Maze, SD rats = Sprague-Dawley rats.

Previous in vivo and in vitro experiments showed that administration of 1-BP evidently impaired spatial learning and memory abilities of rats.^26^ In this study, a MWM test was used to verify the successful establishment of animal model for 1-BP intoxication. Prior to the MWM test, all rats in each group could successfully swim to the platform, and the escape latency time, swimming distance, and swimming speed were measured to evaluate memory impair and spatial learning deficits in the following hidden platform test. Compared with the animals in the control group, SD rats treated with 1-BP spent more time (Figure 1A) and swam longer distance (Figure 1B) before landing on the hidden platform with a comparable swimming speed (Figure 1C), which was markedly mitigated by the pretreatment with melatonin in a concentration-dependent manner (Figure 1). To verify the therapeutic effect of melatonin in the treatment of 1-BP intoxication, 5 different dose of melatonin was intravenously given as described in methods section, and we found that treatment of melatonin significantly ameliorated 1-BP caused impairment of memory ability and spatial learning, as evidenced by its ability to reduce the time and distance for rats to find the hidden platform compared with those exposed to 1-BP in a concentration-dependent manner (Figure 1). Furthermore, the results of the probe test demonstrated that exposure to 1-BP caused the impairment of spatial memory, and all rats treated with 1-BP exhibited significantly reduced number of times they cross the former target platform area, compared with the control group (Figure 2), which was markedly mitigated by the treatment with melatonin in a concentration-dependent manner (Figure 2).

**FIGURE 3:**
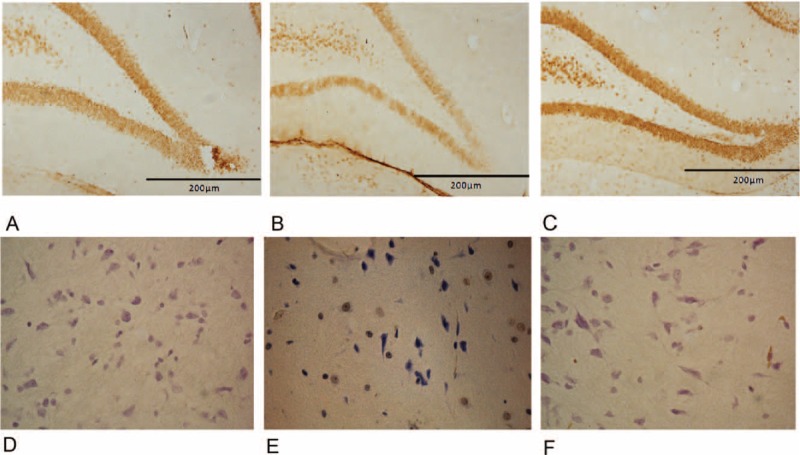
Effects of melatonin on 1-bromopropane (1-BP) induced hippocampal histopathological changes (Neun staining, A: control group, B: 1-BP-treated group; C: 1-BP and melatonin-treated group). Effects of melatonin on 1-bromopropane (1-BP) induced neuron apoptosis in hippocampal (TUNEL assay, A: control group, B: 1-BP-treated group; C: 1-BP and 10 mg/kg BW melatonin-treated group). 1-BP = 1-bromopropane.

**FIGURE 4:**
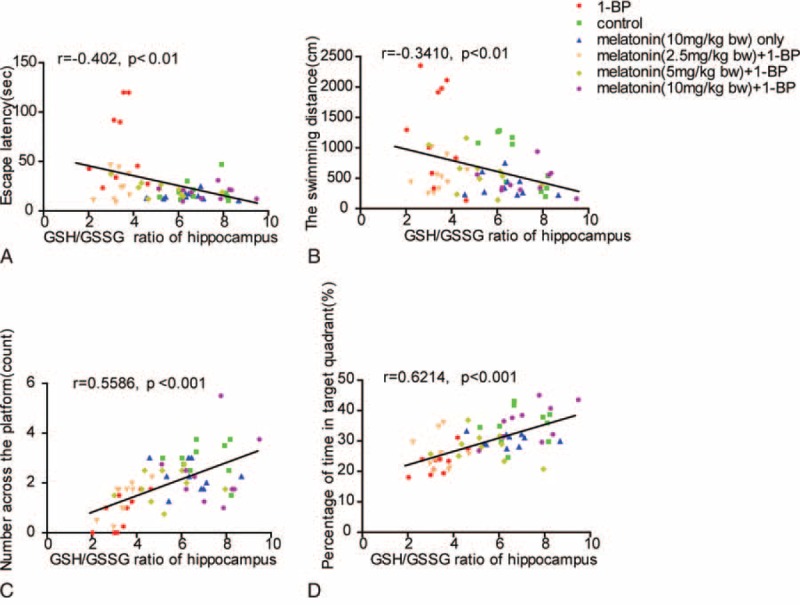
Memory impairment was significantly positively correlated with the GSH/GSSG ratio reduction in hippocampus. The GSH/GSSG ratio of hippocampus displayed significantly negative correlations with the escape latency (A) and the swimming distance (B), and positive correlation with the number across the platform (C) and the percentage of time in target quadrant (D). Correlation analysis was determined by the Spearman correlation coefficient.

**FIGURE 5:**
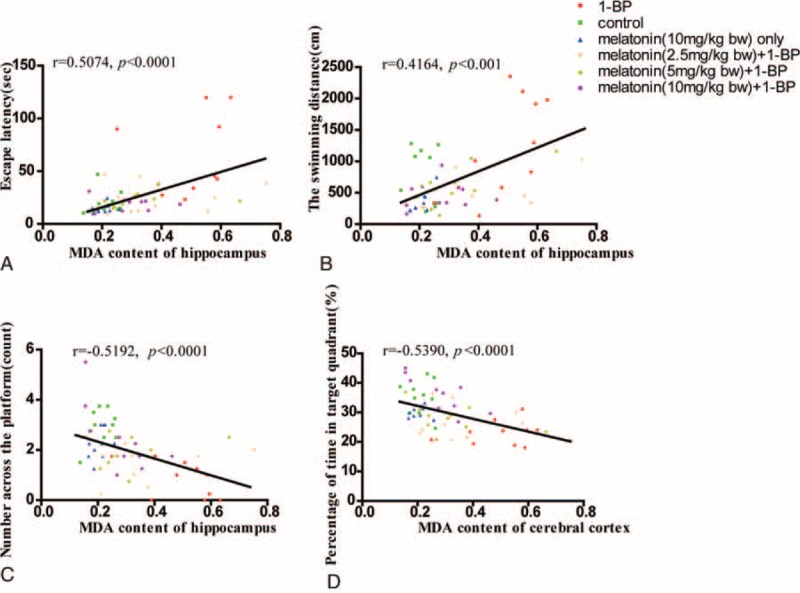
Memory impairment was significantly positively correlated with MDA content increase in hippocampus. MDA content of hippocampus displayed significantly positive correlations with the escape latency (A) and the swimming distance (B), and negative correlation with the number across the platform (C) and the percentage of time in target quadrant (D). Correlation analysis was determined by the Spearman correlation coefficient. MDA = malondialdehyde.

3. Treatment with melatonin restored neuron loss in brains of SD rats exposed to 1-BP Hippocampus has an important role in cognitive function. Therefore, the numbers of neurons in this part were estimated to evaluate the effect of 1-BP on the survival of neurons in Hippocampus. It has been reported that the impairment of memory ability and spatial learning could be resulted from loss of neuron,^27^ we next examined the status of neuron loss in each group. The brains were harvested, sectioned, and stained with Neun to count the neurons in each group. As indicated in Figure 3 1-BP induced notable decrease in neuron population in hippocampus, and such neuron loss was immunohistochemistry visualized in Figure 3, and melatonin pretreatment attenuated those changes in brain (Figure 3). In addition, we determined the apoptosis status of each above group and found that 1-BP induced increase in apoptosis of neurons in hippocampus, and induced apoptosis was evidently attenuated by melatonin treatment (Figure 3).

4. The GSH/GSSG ratio reduction in hippocampus was significantly positively correlated with impairment of memory ability and spatial learning. To further identify the molecular mechanism of the impairment of memory ability and spatial learning, we determined the brain redox status by measuring several important indexes about oxidative stress in hippocampus, and correlations of cognitive performances in the MWM test with the GSH/GSSG ratio of hippocampus were assessed. As shown in Figure 4, the GSH/GSSG ratio of hippocampus was significantly negatively correlated with escape latency and distance traveled, and positively with percentage of time spent in target quadrant and number of platform crossing. Furthermore, another important oxidative radical, MDA, were determined in all experimental animals and its correlation with cognitive performances in the MWM test was also evaluated. As shown in Figure 5, MDA content of hippocampus was significantly positively correlated with escape latency and distance traveled, and negatively with percentage of time spent in target quadrant and number of platform crossing, indicating that there was positive correlation between redox status and cognitive deficits induced by 1-BP.

5. Treatment with melatonin attenuated 1-BP induced oxidative stress in brain.

**FIGURE 6:**
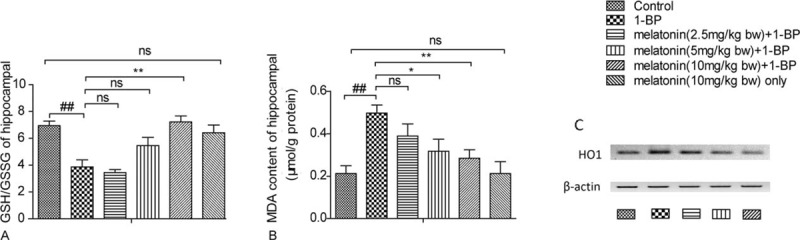
Effects of melatonin on 1-bromopropane(1-BP) induced lipid oxidation stress in cerebral cortex of SD rats. Data were shown as mean ± SEM (n = 10). (A) The GSH/GSSG ratio of cerebral cortex in SD rats. (B) The malondialdehyde (MDA) measurement of cortex in SD rats. (C)The expression of HO1 was notably upregulated upon exposure to 1-BP and was attenuated by the treatment of melatonin. ∗*P* < 0.05, ∗∗*P* < 0.01, ^ns^*P* > 0.05 versus 1-BP group£»^##^*P* < 0.01 versus control group. 1-BP = 1-bromopropane, MDA = malondialdehyde, SD rats = Sprague-Dawley rats.

As shown in Figure 6A, the GSH/GSSG ratio was proportionately decreased in the rats exposed to 1-BP (Figure 6), and administration of melatonin restored the level of the GSH/GSSG ratio. Meanwhile, MDA, the level of lipid peroxidation product, was significantly increased upon exposed to 1-BP, which was significantly attenuated by melatonin pretreatment, indicating that administration of 1-BP could interfere with redox homeostasis of brain in rat, and such 1-BP-induced biomedical changes were reversed by treatment with melatonin. Furthermore, we determined the expression of HO1 using western blot analysis. As shown in Figure 6C, the expression of HO1 was notably upregulated upon exposure to 1-BP and was attenuated by the treatment of melatonin.

## DISCUSSION

In this study, we confirmed that impairment of memory ability and spatial learning in rat exposed to 1-BP, which could be attributed to the loss of neuron in hippo possibly caused by compromised redox homeostasis in brain. Furthermore, the rats exposed to 1-BP was treated with melatonin, and we found administration of melatonin effectively attenuated 1-BP induced impairment of memory ability and spatial learning, and restored the increased MDA and exhausted GSH caused by 1-BP intoxication. Known as a major intracellular antioxidant, GSH is an electron donor to scavenge peroxides catalyzed by GSH-Px at the cost of its becoming oxidized to GSSG—an oxidation form GSH, and therefore plays an essential role in cellular defense against ROS.^28^ GSH interacts nonenzymatically with radicals, and loss of GSH undoubtedly breaks down the redox homeostasis of the brain. The study associated with the disposition and metabolism of 1-BP conducted with murine animals showed that highly active oxidative metabolites were generated in 1-BP intoxication. Other studies have reported the loss of cerebral neurons^29^ and GSH in liver^30^ in rats exposed to 1-BP. GSH produces in vivo from GSSG reduction at the cost of NADPH and synthesis de novo from glycine, cysteine, and glutamate by a range of actions of glutathione synthetase (GS) and -glutamylcysteine synthetase (GCS) in a battery of 6-enzyme-catalyzed reactions.^31^ It has been shown that GSH content in brain is maintained primarily by regeneration from GSSG reduction because of the presence of blood–brain barrier.^28^ In this study, we found that the GSH/GSSG ratio of hippocampus was significantly negatively correlated with escape latency and distance traveled, and positively with percentage of time spent in target quadrant and number of platform crossing. MDA, specifically derived from lipid peroxidation, is abundantly present in brain. It is another highly active electrophilic aldehyde. MDA interacts with amines that contain head groups of phospholipids including phosphatidylethanolamine or phosphatidylserine or with lysine residues of proteins.^32^ MDA is frequently used as a marker for peroxidation of lipid, and also a nonspecific marker for membrane lipid peroxidation.^33^ In this study, MDA content of hippocampus was found to be significantly positively correlated with escape latency and distance traveled, and negatively with percentage of time spent in target quadrant and number of platform crossing, indicating that there was positive correlation between redox status and cognitive deficits induced by 1-BP, and the impairment of memory ability caused by exposure to 1-BP could be directly attributed to the imbalance of redox status.

Melatonin is a potent antioxidant and commonly used as a protective agent to protect against ROS-related damage, which together with the previous report that administration of melatonin prevented 1-BP-induced cell damage directly caused by oxidative attack ,^34^ led us to the idea that melatonin treatment may prevents the brain from 1-BP intoxication. At least 2 mechanisms are involved in the antioxidative properties of melatonin.^35^ On one hand, melatonin directly exhibits antioxidative function via eliminating free radicals and suppressing free radical production. Moreover, melatonin changes the activities of antioxidative enzymes, which enhance the endogenous antioxidative defense ability of the organism. Undoubtedly, 1 significant feature of melatonin in its protection against oxidative stress via eliminating free radical is the direct antioxidative role. In fact, each melatonin molecule eliminates two _OH.^36^ Different from some other well-determined antioxidants, melatonin is amphiphilic; enabling it to decrease free radical-mediated injury in either queous subcellular compartments or the lipid. Melatonin can scavenge peroxyl radical (LOO_), superoxide anion radical (O2-_), singlet oxygen (1O2), and hydrogen peroxide (H_2_O_2_).^37^ In this study, we showed that the GSH/GSSG ratio was proportionately decreased in the rats exposed to 1-BP, and administration of melatonin restored the level of the GSH/GSSG ratio. Meanwhile, MDA, the level of lipid peroxidation product, was significantly increased upon exposed to 1-BP, which was significantly attenuated by melatonin pretreatment, indicating that administration of 1-BP could interfere with redox homeostasis of brain in rat and such 1-BP-induced biomedical changes were reversed by treatment with melatonin.

Although we successfully established the animal model of intoxication by intragastrical administration of 1-BP, and 1-BP-induced neurotoxicity could be attenuated by the treatment with melatonin, such therapeutic effects need to be confirmed in other animal models, and the clinical efficacy and adverse effects of melatonin need to be evaluated in human subjects. In addition, the molecular mechanism of therapeutic effects of melatonin needs to be further explored beyond its ability to scavenge and attenuate the damage of superoxide.

Taken together, the data of the present study showed that exposure to 1-BP led to impairment of memory ability and spatial learning in rat, resulted from the loss of neuron in hippo possibly caused by compromised redox homeostasis in brain. Additionally, administration of melatonin effectively attenuated 1-BP-induced cognitive impairment, and restored the increased MDA and exhausted GSH caused by 1-BP intoxication. The effects of melatonin in preventing 1-BP-induced cognitive impairment may be attributed to its ability to scavenge elevated ROS.
